# Case Report: Identification of a *De novo C19orf12* Variant in a Patient With Mitochondrial Membrane Protein–Associated Neurodegeneration

**DOI:** 10.3389/fgene.2022.852374

**Published:** 2022-03-30

**Authors:** Yue Yang, Shijie Zhang, Wenming Yang, Taohua Wei, Wenjie Hao, Ting Cheng, Jiuxiang Wang, Wei Dong, Nannan Qian

**Affiliations:** ^1^ Graduate School of Anhui University of Chinese Medicine, Hefei, China; ^2^ The First Affiliated Hospital of Anhui University of Chinese Medicine, Hefei, China; ^3^ Xin’an Medical Education Ministry Key Laboratory, Hefei, China; ^4^ Clinical School of Anhui Medical University, Hefei, China

**Keywords:** mitochondrial membrane protein–associated neurodegeneration, *C19orf12*, whole-exome sequencing, iron accumulation, *de novo* variant

## Abstract

**Background:** Mitochondrial membrane protein–associated neurodegeneration (MPAN) mostly arises as an autosomal recessive disease and is caused by variants in the chromosome 19 open reading frame 12 (*C19orf12*) gene. However, a few *C19orf12* monoallelic truncating *de novo* variants have been reported and segregated as autosomal dominant traits in some cases.

**Methods:** We performed whole-exome sequencing and analyzed genes related to neurodegeneration associated with brain iron accumulation for pathogenic variants. The identified variants were confirmed by Sanger sequencing and tested using *in silico* tools.

**Results:** The patient had an onset of depression at the age of 22 years, which rapidly progressed to severe dystonia, dementia, and bladder and bowel incontinence. Neuroimaging showed hypointensity in the substantia nigra and the globus pallidum, with additional frontotemporal atrophy. Genetic analysis revealed a single complex *de novo* variant [c.336_338delinsCACA (p.Trp112CysfsTer40)] in the *C19orf12* gene.

**Conclusion:** This study enriches the genetic spectrum and clinical features of *C19orf12* variants and provides additional evidence of the variable inheritance pattern of MPAN.

## Introduction

Mitochondrial membrane protein–associated neurodegeneration (MPAN; OMIM: 614298) is a subtype of neurodegeneration with brain iron accumulation (NBIA) characterized by dystonia, spastic paraparesis with muscle weakness, cognitive decline progressing to dementia, neuropsychiatric symptoms, and optic atrophy (Olgiati et al., 2017). Brain magnetic resonance imaging (MRI) showed an excessive accumulation of iron in the basal ganglia and substantia nigra of an NBIA patient (Svetel et al., 2021). Usually, chromosome 19 open reading frame 12 (*C19orf12*) displays an entirely autosomal recessive pattern of inheritance in well-characterized cases of MPAN (Hartig et al., 2011); however, a few apparent monoallelic truncating variants in *C19orf12* exon 3 have been reported (Hogarth et al., 2013; Deutschländer et al., 2017; Monfrini et al., 2018).

This report describes a female MPAN patient experiencing onset of depression and identified with a single complex *de novo* variant [c.336_338delinsCACA (p.Trp112CysfsTer40)] in *C19orf12*. This case broadens the mutation spectrum and clinical phenotypes associated with *C19orf12* variants and provides additional evidence of the variable inheritance pattern of MPAN.

## Case Presentation

**FIGURE 1:**
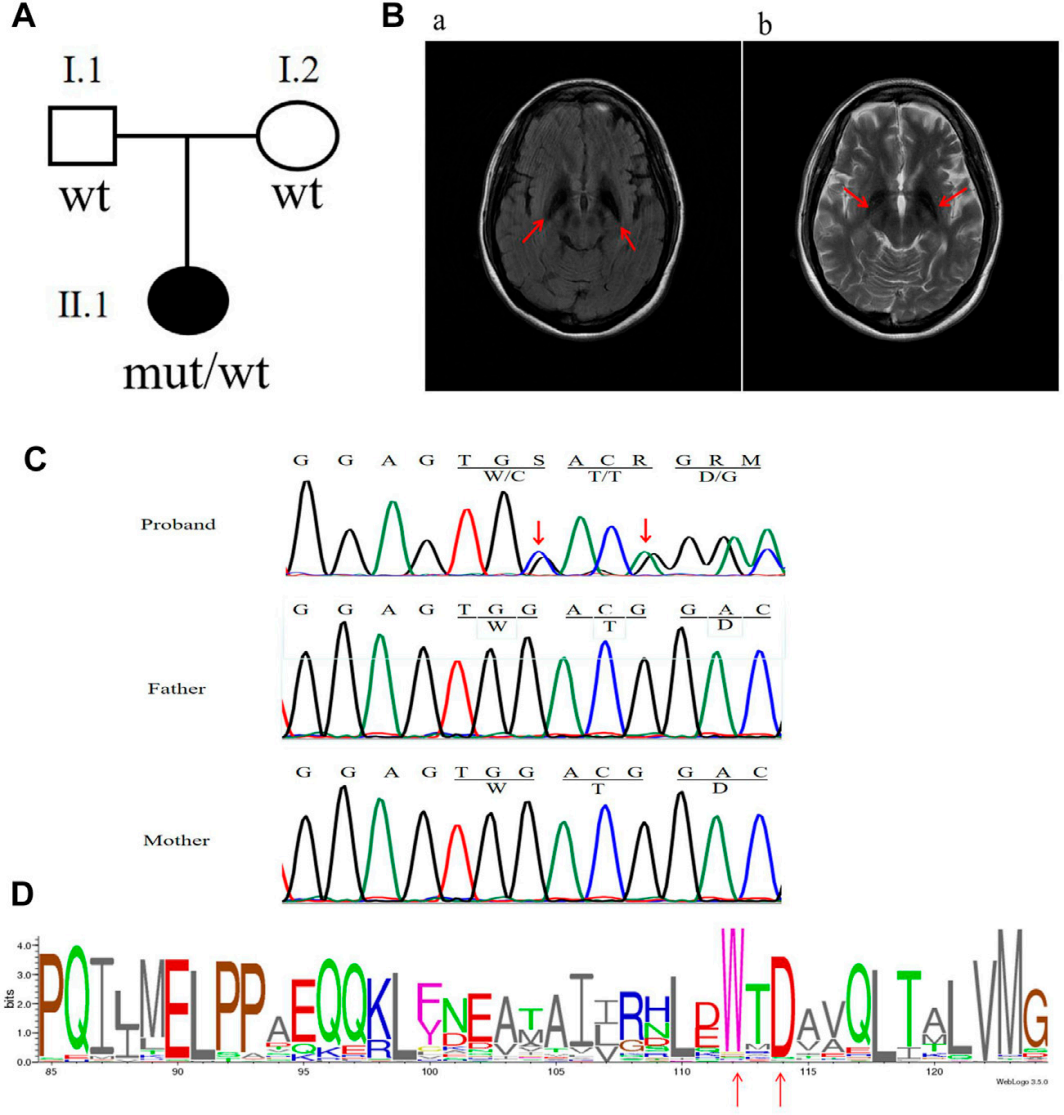
Clinical characteristics and mutation analysis of the family in this study. **(A)** Pedigree of the family in this study. Black symbol denotes the proband. **(B)** Brain MRI results: red arrows show hypointensity in the substantia nigra and globus pallidum along with additional frontotemporal atrophy. **(C)** Sanger sequencing results of the *C19orf12* gene: red arrows indicate the locations of the mutations. **(D)** *C19orf12* variants in the MPAN proband and frequency diagram of protein mutations. The locations of the W112 and D114 sites are indicated *via* red arrows.

The patient was born to a non-consanguineously married couple, met the normal developmental milestones, and had an unremarkable family history (Figure 1A). At the age of 22 years, she presented with a depressed mood and was referred to a psychiatrist. She was subsequently diagnosed with depression; however, antidepressants were ineffective. At 23, she began complaining of a mild tremor in both upper limbs and had difficulty with fine motor skills. A computed tomography scan of the brain showed hyperdensity in the bilateral globus pallidum. At 24, she manifested with gait imbalance, slowed movements, and frequent falls. The symptoms were progressive, and by 26 years, she presented with cognitive decline, dystonia, rigidity, and spasticity. There were no seizures or optic atrophy, and a neurological examination showed moderate dysarthria, cervical dystonia, severe upper limb tremors, spastic limb hypertonia and muscle weakness, patellar hyperreflexia, and bilateral Babinski sign. An MRI of the brain on T2WI and FLAIR showed hypointensity in the substantia nigra and globus pallidum and hyperintense streaking of the medial medullary lamina between the globus pallidum internus and externus (Figure 1B). After a 2-year follow-up, she was immobile and non-verbal, developed obvious bladder and bowel incontinence, was in a wheelchair, and was unable to perform activities of daily living.

## Materials and Methods

### Ethical Compliance

This study was approved by the Medical Ethics Committee of The First Affiliated Hospital of Anhui University of Chinese Medicine (Hefei, China). Informed written consent was obtained from all participants.

### Whole-Exome Sequencing and Validation

Genomic DNA of the patient and her parents was extracted from EDTA-anticoagulated blood, whole-exome sequencing was performed, and genes related to NBIA were analyzed for pathogenic variants. The identified variants were confirmed by Sanger sequencing. To determine whether *C19orf12* variants were located in two different alleles, a fragment containing the two variants was cloned into the pEGFP-C1 vector, and 10 different clones were sent for sequencing. The primer sequences are shown in Supplementary Table S1.

### Variant Interpretation

*C19orf12* variants were compared with the list of reported pathogenic variants in the Human Gene Mutation Database ( http://www.hgmd.cf.ac.uk/ac/gene.php), gnomAD (v3.1.1; http://gnomad-sg.org/), and Ensembl Blast/BLAT (http://asia.ensembl.org/index.html). The pathogenicity of the variants are shown in Supplementary Table S2, which was assessed with SIFT (http://provean.jcvi.org/protein_batch_submit.php), PolyPhen-2 (http://genetics.bwh.harvard.edu/pph2/), and MutationTaster (https://www.mutationtaster.org/). Homology comparisons were conducted by VarSite (https://www.ebi.ac.uk/thornton-srv/databases/cgi-bin/VarSite/GetPage.pl?home=TRUE) to explore whether the mutated region was conserved.

## Results

The analysis of whole-exome sequencing data is shown in Supplementary Table S3, which identified two novel variants [c.336G > C (p.W112C) and c.338_339insA (p.D114Gfs*38)] in *C19orf12* (NM_001031726) that were not present in gnomAD. Furthermore, neither of the variants were found in the parents of the patient (Figure 1C). VarSite showed that the variants were located in the highly conserved region of the *C19orf12* gene across multiple species (Figure 1D). Supplementary Table S2 shows additional evidence for the pathogenicity of the two novel variations. According to the American College of Medical Genetics and Genomics guidelines, the frameshift variant c.338-339insA and missense variant c.336G > C can be classified as pathogenic (PVS1, PS2, PM2, PP3) and likely pathogenic (PS2, PM2, PP3), respectively.

Sanger sequencing confirmed that the two variants were located in the same allele (Supplementary Figure S1). However, given the unlikelihood that two *de novo* variants occur on the same allele, we treated these variants as a single complex *de novo* variant [c.336_338delinsCACA (p.Trp112CysfsTer40)].

## Discussion

MPAN is a classical form of NBIA and characterized by juvenile-onset and as a slowly progressive phenotype with dystonia, speech disturbances, cognitive decline, optic atrophy, and spastic paraparesis. However, psychiatric disturbances are relatively rare (Hartig et al., 2013; Schulte et al., 2013). Brain MRI results for MPAN are characterized by hypointensity in the substantia nigra and globus palladium (Hogarth et al., 2013), and neuroimaging confirmed hyperintense streaking in the medial medullary lamina, which is typical of MPAN and present in this case. Moreover, we found that additional frontotemporal atrophy was also present, which is an imaging feature associated with the end stages in MPAN individuals. In the present case, MPAN onset included depression at age 22, which progressed rapidly to severe dystonia, dementia, bladder and bowel incontinence, and brain atrophy within 4 years. Additionally, this patient did not have optic atrophy or blurred vision as compared with reports from other studies (de Vries et al., 2021; Langwinska-Wosko et al., 2016).

C19orf12 is a mitochondrial protein with a complex isoform involved in maintaining lipid homeostasis and membrane remodeling, with mutated variants capable of inducing oxidative stress and causing neuroinflammation (Hinarejos et al., 2020). However, it remains unclear how *C19orf12* variants induce iron accumulation and orchestrate the MPAN phenotype. Ferroptosis may be a novel theory for the pathophysiologic mechanism of MPAN and might be related to lipid peroxidation and mitochondrial perturbation (Wang et al., 2019). To date, >50 *C19orf12* variants have been reported in MPAN cases according to the Human Gene Mutation Database, and abundant evidence supports an autosomal recessive pattern. However, Monfrini et al. (2018) proposed a dominant negative effect associated with a *C19orf12* variant in a patient with MPAN who carried a *de novo* heterozygous mutation. Additionally, Gregory et al. (2019) provided evidence of the molecular mechanism, suggesting the presence of a functional region in C19orf12 that contained a glycine zipper motif that could have a multimerization function (Gagliardi et al., 2015). The *C19orf12* variant resulting in a protein truncated after amino acid 79 may retain the ability to multimerize with the protein from the wild-type allele; however, this version of the protein may still cause damage or induce degradation of the resulting protein complex, resulting in loss of function (Gregory et al., 2019). However, this would not explain a protein variant truncated after amino acid 76 (p.Met76Thrfs*3; NM_00103176.3) in patients with autosomal dominant MPAN. Thus, Rickman et al. (2021) considered “haploinsufficiency of isoform 3” as a potential mechanism of monoallelic MPAN, which results in a protein truncated after amino acid 75 and lacking the putative transmembrane region. Other studies subsequently confirmed an autosomal dominant mode of inheritance in MPAN (Fraser et al., 2021; Rickman et al., 2021).

A literature review focusing on age at onset, clinical features, and *C19orf12* variant status [homozygous/compound heterozygous (Supplementary Table S1) and heterozygous patients (Table 1)] in MPAN cases indicated that 57 of 70 of cases presented with clinical signs of neurodegeneration before age 18. Moreover, we performed the analysis (Mann–Whitney test) and revealed homozygous/compound heterozygous *C19orf12* mutations associated with MPAN cases had an earlier age at onset than heterozygous cases [biallelic vs. monoallelic: 9 ± 4 (range: 3–29 years) vs. 19 ± 16 (range: 1–55 years), *p* = 0.042]. Independent of the inheritance pattern, most MPAN cases are clinically similar, with the most common presenting symptoms being cognitive decline, gait difficulties, and optic atrophy, which occurred in 38, 34, and 21 of 70 cases, respectively.

**TABLE 1:** Heterozygous *C19orf12* mutations associated with MPAN cases in the medical literature.

Gene variant	Age at onset	Major features	References
c.297insGCTC (p.L99fs102)	7–8 years	Behavioral disturbances, cognitive decline, optic atrophy, bradykinetic-rigid syndrome	Panteghini et al. (2012)
c.244A>T (p.Lys82*)	10 years	Visual failure, progressive dystonia, gait impairment	Gagliardi et al. (2015)
c.265_266delAT *de novo* (p.M89Gfs*12)	5 years	Progressive imbalanced gait with rigidity, dystonia	Monfrini et al. (2018)
c.227_237del11 (p.Met76Thrfs*3)	55 years	Cognitive decline, Parkinsonism	Gregory et al. (2019)
c.227_237del11 (p.Met76Thrfs*3)	38 years	Hypomimia, hypophonia, rigidity, bradykinesia	Gregory et al. (2019)
c.227_237del11 (p.Met76Thrfs*3)	38 years	Depression, gait changes, cognitive decline	Gregory et al. (2019)
c.227_237del11 (p.Met76Thrfs*3)	34 years	Gait imbalance, motor slowness, tremors, anxiety	Gregory et al. (2019)
c.336G>A (p.Trp112*)	30 years	Parkinsonism, cognitive decline, psychiatric symptoms	Gregory et al. (2019)
c.336G>A (p.Trp112*)	55 years	Personality changes, slowed movements, cognitive decline	Gregory et al. (2019)
c.278delC (p.Pro93Leufs*26)	18 years	Optic atrophy, progressive Parkinsonism, cognitive decline	Gregory et al. (2019)
c.256C>T(p.Gln86*)	12 years	Gait changes, wheelchair at 18 years, optic atrophy, cognitive decline	Gregory et al. (2019)
c.278dupC (p.Pro93Profs*8)	9 years	Cognitive decline, optic atrophy, dystonia, dysarthria	Gregory et al. (2019)
c.357dupG(p.Ala120Glyfs*32)	29 years	Neuropsychiatric changes, Parkinsonism, cognitive decline	Gregory et al. (2019)
c.279delT (p.Ala94Profs)	9 years	Falling, poor school performance, dysarthria	Gregory et al. (2019)
c.300delT (p.Phe100Leufs*19)	5 years	Gait changes, optic atrophy, spastic paraparesis, cognitive decline	Gregory et al. (2019)
c.268G>T (p.Glu90*)	22 years	Gait changes, depression, mild dystonia, dysarthria	Gregory et al. (2019)
c.279_282del TGCC *de novo* (p.Ala94Serfs*24)	4 years	Developmental delay, spasticity, dystonia, disinhibited personality	Gregory et al. (2019)
c.349C>T (p.Gln117*)	18 months	Dystonia, lower limb spasticity, hearing loss	Gregory et al. (2019)
c.238C>T (p.Gln80*)	10 years	Spastic tetraparesis, optic disc pallor, dysphagia	Gregory et al. (2019)
c.238C>T *de novo* (p.Gln80*)	5 years	Gait disturbance, optic atrophy, neuropsychiatric symptoms	Gregory et al. (2019)
c.278delG *de novo* (p.Pro93Leufs*26)	20 years	Cognitive decline, gait instability, frequent falls	Rickman et al. (2021)

**FIGURE 2:**
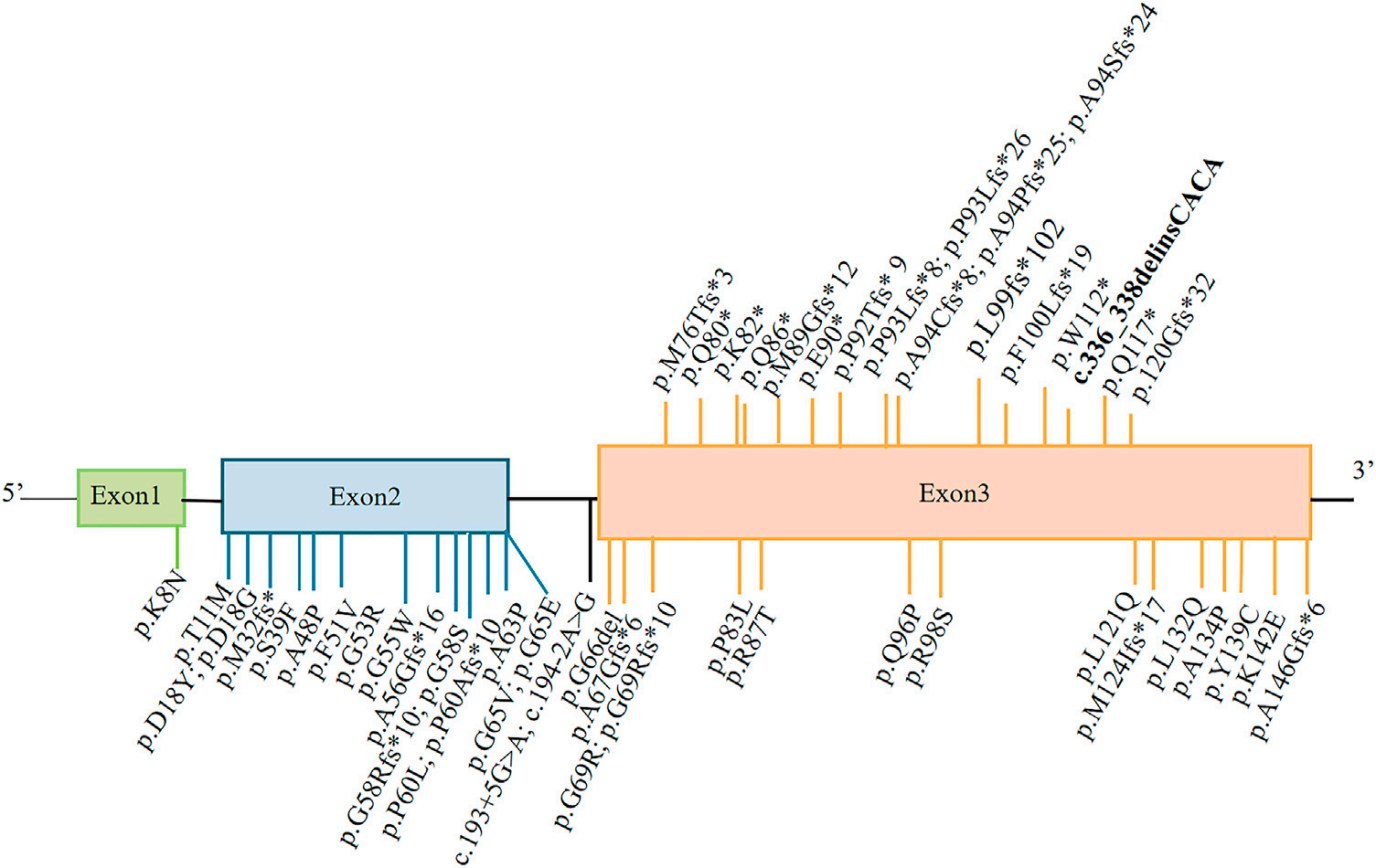
Representation of MPAN-related *C19orf12* variants. The variants shown above the gene structure are monoallelic, and those shown below are biallelic. The variant reported in this study is shown in bold.

As of December 2021, 17 monoallelic truncating variants have been described in patients with MPAN (Figure 2). In this case report, genetic analysis indicated a single complex variant of c.336_338delinsCACA (p.Trp112CysfsTer40) in *C19orf12*, which represents a *de novo* occurrence. This *C19orf12* variant occurs at position 112 and subsequent codons, resulting in a series of 40 amino acid substitutions and causing early translation interruption during protein translation. Therefore, abnormal protein products are likely to escape nonsense-mediated mRNA decay and may have a significant negative impact on normally translated C19orf12 proteins.

In summary, we identified a single complex *de novo* variant of *C19orf12* [c.336_338delinsCACA (p.Trp112CysfsTer40)] in an MPAN patient with a history of onset of depression that rapidly progressed to severe dystonia, dementia, and bladder and bowel incontinence. Additionally, a review of previously reported MPAN cases supported the novelty of this variant and provided additional evidence of the variable inheritance pattern associated with MPAN.

## Data Availability

The datasets for this article are not publicly available due to concerns regarding participant/patient anonymity. Requests to access the datasets should be directed to the corresponding author.

## References

[B1] de Vries R. J. Hellebrekers D. M. E. I. Reneman L. Verhamme C. Smeets H. J. M. van Maarle M. C. (2021). Distal Muscle Weakness and Optic Atrophy without central Nervous System Involvement in a Patient with a Homozygous Missense Mutation in the C19ORF12-Gene. Clin. Neurol. Neurosurg. 206, 106637. 10.1016/j.clineuro.2021.106637 34022688

[B2] Deschauer M. Gaul C. Behrmann C. Prokisch H. Zierz S. Haack T. B. (2012). C19orf12 Mutations in Neurodegeneration with Brain Iron Accumulation Mimicking Juvenile Amyotrophic Lateral Sclerosis. J. Neurol. 259, 2434–2439. 10.1007/s00415-012-6521-7 22584950

[B3] Deutschländer A. Konno T. Ross O. A. (2017). Mitochondrial Membrane Protein-Associated Neurodegeneration. Parkinsonism Relat. Disord. 39, 1–3. 10.1016/j.parkreldis.2017.03.014 28359667

[B4] Dušek P. Školoudík D. Roth J. Dušek P. (2018). Mitochondrial Membrane Protein-Associated Neurodegeneration: a Case Report and Literature Review. Neurocase 24, 161–165. 10.1080/13554794.2018.1506038 30088953

[B5] Fraser S. Koenig M. Farach L. Mancias P. Mowrey K. (2021). A De Novo Case of Autosomal Dominant Mitochondrial Membrane Protein‐associated Neurodegeneration. Mol. Genet. Genomic Med. 9, e1706. 10.1002/mgg3.1706 34041867 PMC8372066

[B6] Gagliardi M. Annesi G. Lesca G. Broussolle E. Iannello G. Vaiti V. (2015). C19orf12 Gene Mutations in Patients with Neurodegeneration with Brain Iron Accumulation. Parkinsonism Relat. Disord. 21, 813–816. 10.1016/j.parkreldis.2015.04.009 25962551

[B7] Gowda V. K. Patil A. Srinivasan V. M. Kathrani N. (2019). Mitochondrial Membrane Protein Associated Neurodegeneration (MPAN) with a Novel C19orf12 Mutation in the First Decade of Life. Indian J. Pediatr. 86, 746–748. 10.1007/s12098-019-02903-w 30825065

[B8] Gregory A. Lotia M. Jeong S. Y. Fox R. Zhen D. Sanford L. (2019). Autosomal Dominant Mitochondrial Membrane Protein‐associated Neurodegeneration (MPAN). Mol. Genet. Genomic Med. 7. 10.1002/mgg3.736 PMC6625130 31087512

[B9] Hartig M. B. Iuso A. Haack T. Kmiec T. Jurkiewicz E. Heim K. (2011). Absence of an Orphan Mitochondrial Protein, C19orf12, Causes a Distinct Clinical Subtype of Neurodegeneration with Brain Iron Accumulation. Am. J. Hum. Genet. 89, 543–550. 10.1016/j.ajhg.2011.09.007 21981780 PMC3188837

[B10] Hartig M. Prokisch H. Meitinger T. Klopstock T. (2013). Mitochondrial Membrane Protein-Associated Neurodegeneration (MPAN). Int. Rev. Neurobiol. 110, 73–84. 10.1016/B978-0-12-410502-7.00004-1 24209434

[B11] Hinarejos I. Machuca C. Sancho P. Espinós C. (2020). Mitochondrial Dysfunction, Oxidative Stress and Neuroinflammation in Neurodegeneration with Brain Iron Accumulation (NBIA). Antioxidants 9, 1020. 10.3390/antiox9101020 PMC7589120 33092153

[B12] Hogarth P. Gregory A. Kruer M. C. Sanford L. Wagoner W. Natowicz M. R. (2013). New NBIA Subtype: Genetic, Clinical, Pathologic, and Radiographic Features of Mpan. Neurology 80, 268–275. 10.1212/WNL.0b013e31827e07be 23269600 PMC3589182

[B13] Incecik F. Herguner O. Bisgin A. (2019). Mitochondrial Membrane Protein-Associated Neurodegeneration: a Case Series of Six Children. Ann. Indian Acad. Neurol. 23, 802–804. 10.4103/aian.AIAN_268_19 33688131 PMC7900730

[B14] Kasapkara Ç. S. Tümer L. Gregory A. Ezgü F. İnci A. Derinkuyu B. E. (2019). A New NBIA Patient from Turkey with Homozygous C19ORF12 Mutation. Acta Neurol. Belg. 119, 623–625. 10.1007/s13760-018-1026-5 30298423 PMC7556727

[B15] Langwinska-Wosko E. Skowronska M. Kmiec T. Czlonkowska A. (2016). Retinal and Optic Nerve Abnormalities in Neurodegeneration Associated with Mutations in C19orf12 (MPAN). J. Neurol. Sci. 370, 237–240. 10.1016/j.jns.2016.09.046 27772766

[B16] Li S. J. Wang L. L. Qin L. Z. Wang X. J. Zhang J. W. Li W. (2019). Pedigree Analysis of C19ORF12 p.Asp18Tyr Mutation in a Family with Mitochondrial Membrane Protein Associated Neurodegeneration. Zhonghua yi xue za zhi 99, 2926–2931. 10.3760/cma.j.issn.0376-2491.2019.37.011 31607023

[B17] Monfrini E. Melzi V. Buongarzone G. Franco G. Ronchi D. Dilena R. (2018). A *De Novo* C19orf12 Heterozygous Mutation in a Patient with MPAN. Parkinsonism Relat. Disord. 48, 109–111. 10.1016/j.parkreldis.2017.12.025 29295770

[B18] Nagarjunakonda S. Daggumati R. Uppala V. Gajula R. Amalakanti S. (2019). A Novel Mutation in Neurodegeneration with Brain Iron Accumulation - A Case Report. Neurol. India 67, 1341–1343. 10.4103/0028-3886.271257 31744972

[B19] Olgiati S. Doğu O. Tufekcioglu Z. Diler Y. Saka E. Gultekin M. (2017). The p.Thr11Met Mutation in C19orf12 Is Frequent Among Adult Turkish Patients with MPAN. Parkinsonism Relat. Disord. 39, 64–70. 10.1016/j.parkreldis.2017.03.012 28347615

[B20] Panteghini C. Zorzi G. Venco P. Dusi S. Reale C. Brunetti D. (2012). C19orf12 and Fa2h Mutations Are Rare in Italian Patients with Neurodegeneration with Brain Iron Accumulation. Semin. Pediatr. Neurol. 19, 75–81. 10.1016/j.spen.2012.03.006 22704260

[B21] Rickman O. J. Salter C. G. Gunning A. C. Fasham J. Voutsina N. Leslie J. S. (2021). Dominant Mitochondrial Membrane Protein-Associated Neurodegeneration (MPAN) Variants Cluster within a Specific C19orf12 Isoform. Parkinsonism Relat. Disord. 82, 84–86. 10.1016/j.parkreldis.2020.10.041 33260061

[B22] Schulte E. C. Claussen M. C. Jochim A. Haack T. Hartig M. Hempel M. (2013). Mitochondrial Membrane Protein Associated Neurodegenration: a Novel Variant of Neurodegeneration with Brain Iron Accumulation. Mov Disord. 28, 224–227. 10.1002/mds.25256 23436634

[B23] Selikhova M. Fedotova E. Wiethoff S. Schottlaender L. V. Klyushnikov S. Illarioshkin S. N. (2017). A 30-year History of MPAN Case from Russia. Clin. Neurol. Neurosurg. 159, 111–113. 10.1016/j.clineuro.2017.05.025 28641177

[B24] Skowronska M. Kmiec T. Kurkowska-Jastrzębska I. Czlonkowska A. (2015). Eye of the Tiger Sign in a 23year Patient with Mitochondrial Membrane Protein Associated Neurodegeneration. J. Neurol. Sci. 352, 110–111. 10.1016/j.jns.2015.03.019 25819119

[B25] Sparber P. Marakhonov A. Filatova A. Sharkova I. Skoblov M. (2018). Novel Case of Neurodegeneration with Brain Iron Accumulation 4 (NBIA4) Caused by a Pathogenic Variant Affecting Splicing. Neurogenetics 19, 257–260. 10.1007/s10048-018-0558-4 30392167

[B26] Svetel M. Dragašević N. Petrović I. Novaković I. Tomić A. Kresojević N. (2021). NBIA Syndromes: a Step Forward from the Previous Knowledge. Neurol. India 69, 1380–1388. 10.4103/0028-3886.329603 34747818

[B27] Tariq H. Butt J. U. R. Houlden H. Naz S. (2019). Are Some C19orf12 Variants Monoallelic for Neurological Disorders? Parkinsonism Relat. Disord. 65, 267–269. 10.1016/j.parkreldis.2019.05.020 31105013

[B28] Wang Z.-B. Liu J.-Y. Xu X.-J. Mao X.-Y. Zhang W. Zhou H.-H. (2019). Neurodegeneration with Brain Iron Accumulation: Insights into the Mitochondria Dysregulation. Biomed. Pharmacother. 118, 109068. 10.1016/j.biopha.2019.109068 31404774

