# Supplementary material for: Case Report: Identification of a De novo C19orf12 Variant in a Patient With Mitochondrial Membrane Protein–Associated Neurodegeneration
Source: Front Genet. 2022 Mar 30;13:852374. doi: 10.3389/fgene.2022.852374 (PMC9006254; doi:10.3389/fgene.2022.852374)
Supplement: Supplementary file 1 [file Table1.docx]

Table 2. Homozygous/compound heterozygous cases in the medical literature

| **Gene variant** | **Age at onset** | **Major features** | **References** |
| --- | --- | --- | --- |
| c.204_214del (homozygous) (p.G69RfsX10) | 6 years | Clumsiness, fatigue, optic atrophy, gait spasticity | (Hartig et al.,2011) |
| c.287A>C (homozygous) (p.Q96P) | 9 years | Optic atrophy, spastic ataxic gait, dysarthria, swallowing difficulties | (Panteghini et al.,2012) |
| c.172G>A (homozygous) (p.G58R) | 13 years | Spasticity, prominent ataxia, dysphonia | (Panteghini et al.,2012) |
| c.32C>T (p.Thr11Met)  c.197-199del3 (p.Gly66del) | 21 years | Difficulties in doing needlework, atrophies of her hands | (Deschauer et al.,2012) |
| c.32C>T (p.Thr11Met)  c.197-199del3 (p.Gly66del) | 3years | Motor and mental delay, impulsive behavior, hyperreflexia, pes cavus | (Deschauer et al.,2012) |
| c.197-199del3 (p.Gly66del)  c.204-214del11 (p.Gly69Argfs*10) | 9 years | Gait difficulties, pes cavus, marked thenar and hypothenar atrophy, frequent falls | (Deschauer et al.,2012) |
| c.32 C>T (p.Thr11Met)  c.204_214del (p.Gly69ArgfsX10) | 7 years | Cognitive impairment, motor axonal neuropathy | (Schulte et al.,2013) |
| c.53 A>G (p.Asp18Gly)  c.395 T>A (p.Leu132Gln) | 3 years | Pyramidal signs, spasticity, optic atrophy, motor axonal neuropathy, psychiatric, cognitive impairment | (Schulte et al.,2013) |
| c.204_214del (homozygous)  (p.Gly69ArgfsX10) | 6years | Pyramidal signs, dysarthria, spasticity | (Schulte et al.,2013) |
| c.204_214del11 (homozygous)  (p.Gly69ArgfsX10) | 9 years | Visual acuity, gait impairment, spastic tetraparesis, severe optic atrophy | (Skowronska et al.,2015) |
| c.436_437insG (homozygous)  (p.Ala146Glyfs*6) | 7 years | Gait difficulties, dystonia, rigidity, spasticity, intellectual decline, psychiatric disturbances | (Gagliardi et al.,2015) |
| c.287A>C (homozygous)(p.Gln96Pro) | 7 years | Visual problems, spastic ataxic gait | (Gagliardi et al.,2015) |
| c.199_199delG (p.Ala67Leufs*6)  c.416A>G (p.Tyr139Cys) | 7 years | Behavioral disturbances, cognitive decline, bradykinetic-rigid syndrome, dystonia, visual failure | (Gagliardi et al.,2015) |
| c.248C>T (p.Pro83Leu)  c.400G>C (p.Ala134Pro) | 9 years | cognitive impairmen, bradykinesia, gait impairment | (Gagliardi et al.,2015) |
| c.204_214del11 (homozygous) (p.Gly69ArgfsX10) | 10 years | Stumbled gait, legs weakness, optic atrophy, ataxic, dysarthric, epileptic seizure | (Selikhova et al.,2017) |
| c.32C>T (p.Thr11Met)  c.205G>A (p.Gly69Arg)  c.424A>G (p.Lys142Glu) | 10 years | Foot dystonia, impaired handwriting, dysarthria, spastic-dystonic gait | (Dušek et al.,2018) |
| c.204_214del11 (p.Gly69Argfs*10)  c.193+5G>A (p.?) | 12 years | Gait impairment, staggering, learning difficulties, dysarthria, enuresis, vision loss | (Sparber et al.,2018) |
| c.171_181delCGGGGGGCTGT  (homozygous) (p.Gly58Argfs*10) | 4 years | Frequent falls, intellectual decline, psychiatric disturbances, axonal neuropathy | (Kasapkara et al.,2019) |
| c.199delG (homozygous) (p.Ala67LeufsTer5) | 8 years | Frequent falls, rigidity, decreased visual acuity, mild ataxia, cognitive impairment | (Tariq et al.,2019) |
| c.163G > T (homozygous) (p.G55 W) | 8 years | Pyramidal signs, dystonia, dysarthria, pale optic disc | (Gowda et al.,2019) |
| (homozygous) (p.F51V) | 12 years | Progressive gait disturbance | (Nagarjunakonda et al.,2019) |
| c.116C>T (p.Ser39Phe)  c.205G>A (p.Gly69Arg) | 11 years | Gait changes, cognitive decline, dystonia | (Gregory et al.,2019) |
| c.204_211del11(homozygous)  (p.Gly69Argfs*10) | 10 years | Tremor, cognitive decline, spastic tetraparesis, optic atrophy | (Gregory et al.,2019) |
| c.204_211del11 (p.Gly69Argfs*10)  c.157G>A (p.Gly53Arg) | 10 years | Gait change, spasticity, optic atrophy | (Gregory et al.,2019) |
| c.248C>T (p.Pro83Leu)  c.400G>C (p.Ala134Pro) | 10 years | Optic atrophy, spasticity, parkinsonism, cognitive decline | (Gregory et al.,2019) |
| c.204_211del11 (p.Gly69Argfs*10)  c.294G>C (p.Arg98Ser) | 29 years | Cognitive decline, expressive dysphasia, parkinsonism | (Gregory et al.,2019) |
| c.204_211del11 (p.Gly69Argfs*10)  c.205G>A (p.Gly69Arg) | 9 | Dysarthria, gait change, dystonia, parkinsonism, incontinence, cognitive decline | (Gregory et al.,2019) |
| c.194delG (homozygous) (p.A67Lfs*6) | 13 years | Gait changes, cognitive decline, incontinanc | (Gregory et al.,2019) |
| c.194delG (homozygous) (p.A67Lfs*6) | 13 years | Gait changes, cognitive decline, dysarthria | (Gregory et al.,2019) |
| c.142G>C (p.Ala48Pro)  c.194−2A>G (p.?) | 10 years | Progressive spasticity, parkinsonism, cognitive decline | (Gregory et al.,2019) |
| c.142G>C (p.Ala48Pro)  c.194−2A>G (P?) | 10 years | Developmental delay, spasticity, gait changes | (Gregory et al.,2019) |
| c.157G>A (p.Gly53Arg)  c.205G>A (p.Gly69Arg) | 8 years | Progressive spasticity, intention tremor, gait changes | (Gregory et al.,2019) |
| c.194G>A (homozygous) (p.Gly65Glu) | 4 years | Psychosis, dystonia, tremor | (Gregory et al.,2019) |
| c.194G>T (p.Gly65Val)  c.179C>T (p.Pro60Leu) | 4 years | Spastic paraparesis, dysarthria, developmental delay | (Gregory et al.,2019) |
| c.194G>T (homozygous) (p.Gly65Val） | 6 years | Optic atrophy, spasticity, cognitive decline | (Gregory et al.,2019) |
| c.204_211del11 (homozygous)  (p.Gly69ArgfsX10) | 3 years | Dystonia, neuropsychiatric changes, cognitive decline, intention tremor | (Gregory et al.,2019) |
| c.194G>A (p.Gly65Glu)  c.400G>C (p.Ala134Pro) | 9 years | Spasticity, optic atrophy, cognitive decline | (Gregory et al.,2019) |
| c.204_211del11 (homozygous)  (p.Gly69Argfs*10) | 6 years | Spastic paraparesis, optic atrophy, dystonia, parkinsonism, cognitive decline | (Gregory et al.,2019) |
| c.171_181del11 (homozygous)  (p.Gly58Argfs*10) | 14 years | Dysarthria, dystonia, incontinence, neuropsychiatric changes | (Gregory et al.,2019) |
| c.194G>Tl ( p.Gly65Va)  c.204_214del11 (p.Gly69Argfs*10 ) | 13 years | Spasticity, motor decline, optic atrophy, cognitive decline, psychiatric changes | (Gregory et al.,2019) |
| c.194G>T (p.Gly65Val )  c.204_214del11 (p.Gly69Argfs*10) | 11 years | Progressive lower extremity spasticity, optic atrophy, cognitive decline | (Gregory et al.,2019) |
| c.94delA (p.Met32fs*）  c.248C>T (p.Pro83Leu) | 10 years | Gait change, optic atrophy, spasticity, dystonia, cognitive decline | (Gregory et al.,2019) |
| c.205G>A (homozygous) (p.Gly69Arg) | 7 years | Gait change, dysarthria, dysphagia, spasticity | (Gregory et al.,2019) |
| c.52G>T (homozygous) (p.Asp18Tyr) | 10 years | Slowed movement, gait change, dysarthria | (Li et al.,2019) |
| c.52G>T (homozygous) (p.Asp18Tyr) | 8 years | Slowed movement, gait change, dysarthria, cognitive decline, neuropsychiatric changes | (Li et al.,2019) |
| c.52G>T (homozygous) (p.Asp18Tyr) | 7 years | Slowed movement, gait change, dysarthria, cognitive decline, psychiatric changes | (Li et al.,2019) |
| c.371_372insT (homozygous) (p.M124Ifs*17) | 10 years | Frequent falls, behavioral disorders, cognitive impairment, dysarthria | (Incecik et al.,2020) |
| c. 166_167insG (homozygous)  (p.Ala56Glys*16) | 10 years | Gait impairment, dystonia, bradykinesia, cognitive impairment, behavioral disturbances, visual failure, spasticity | (Incecik et al.,2020) |
| c. 371_372insT (homozygous) (p.M124Ifs*17) | - | Optic atrophy, dysarthria, dystonia, tremor, behavioral disturbances, cognitive impairment, spastic gait | (Incecik et al.,2020) |
