# Supplementary material for: Case Report: Identification of a De novo C19orf12 Variant in a Patient With Mitochondrial Membrane Protein–Associated Neurodegeneration
Source: Front Genet. 2022 Mar 30;13:852374. doi: 10.3389/fgene.2022.852374 (PMC9006254; doi:10.3389/fgene.2022.852374)
Supplement: Supplementary file 2 [file DataSheet1.ZIP › Supplementary Figure 1.docx]

Supplementary Material


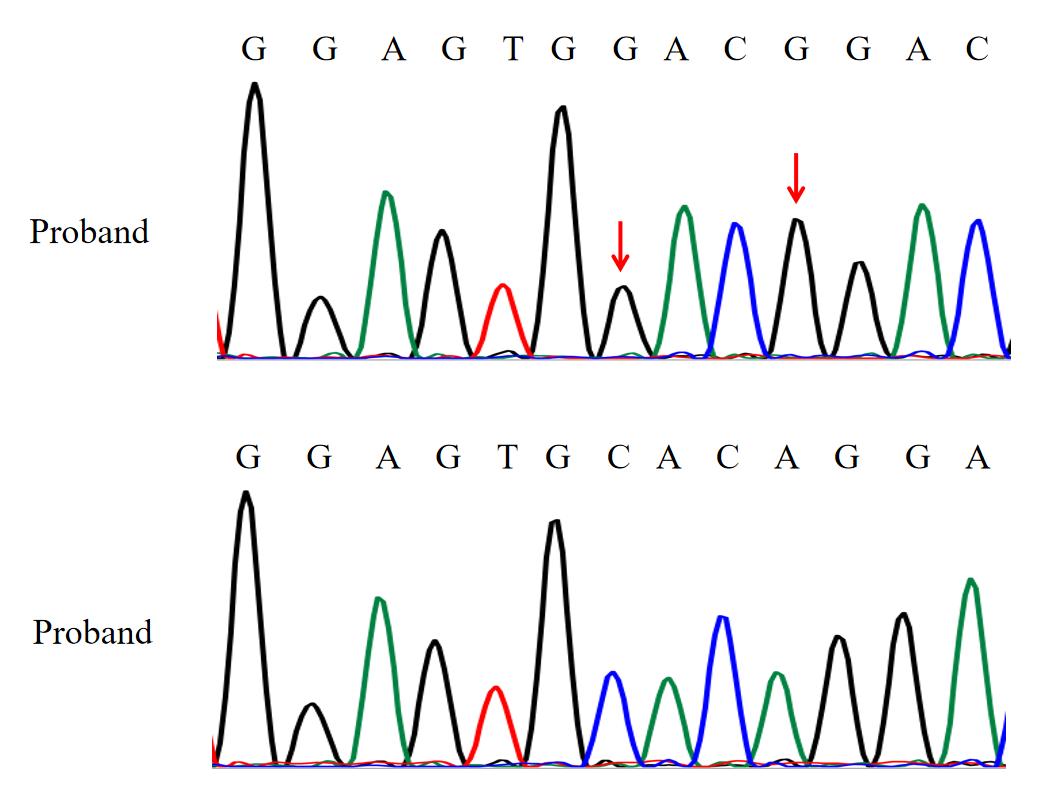


Supplementary Figure 1. Sanger sequencing of the constructed plasmids. The upper sequence presents two mutations(red arrows), and the lower is the wild-type sequence
