# Supplementary material for: Case Report: Identification of a De novo C19orf12 Variant in a Patient With Mitochondrial Membrane Protein–Associated Neurodegeneration
Source: Front Genet. 2022 Mar 30;13:852374. doi: 10.3389/fgene.2022.852374 (PMC9006254; doi:10.3389/fgene.2022.852374)
Supplement: Supplementary file 2 [file DataSheet1.ZIP › Supplementary table 1.docx]

Supplementary Material

**Supplementary table 1. Primers sequences were used in this study.**

| Names | Sequences(5’-3’) |
| --- | --- |
| *C19orf12*-seq-f | AGACCCACTAATTGAACGAT |
| *C19orf12*-seq-r | CAACACATGCTGCTTCATCA |
| *C19orf12*-f-KpnI | GCGggtaccAGACCCACTAATTGAACGAT |
| *C19orf12*-r-BamHI | GCGggatccCAACACATGCTGCTTCATCA |
