# Supplementary material for: Case Report: Identification of a De novo C19orf12 Variant in a Patient With Mitochondrial Membrane Protein–Associated Neurodegeneration
Source: Front Genet. 2022 Mar 30;13:852374. doi: 10.3389/fgene.2022.852374 (PMC9006254; doi:10.3389/fgene.2022.852374)
Supplement: Supplementary file 2 [file DataSheet1.ZIP › Supplementary table 2.docx]

Supplementary Material

**Supplementary Table 2. Evidence supporting the pathogenicity of the two novel variants.**

| ****Variation/Gene**** | **c.336G>C**,** *C19orf12*** | **c.338-339insA**,** *C19orf12*** |
| --- | --- | --- |
| ****Position (Hg38)**** | Chr19:30193742 | chr19:30193739_30193740 |
| ****Transcript ID**** | [NM_001031726](http://www.ncbi.nlm.nih.gov/nuccore/NM_001031726) | [NM_001031726](http://www.ncbi.nlm.nih.gov/nuccore/NM_001031726) |
| ****UniProt Peptide**** | [Q9NSK7](http://www.uniprot.org/uniprot/Q9NSK7) | [Q9NSK7](http://www.uniprot.org/uniprot/Q9NSK7) |
| ****Exon**** | Exon 3 of 3 | Exon 3 of 3 |
| ****Protein**** | p.W112C | p.D114Gfs*38 |
| ****Impact**** | Missense | Frameshift |
| ****ACMG**** | Likely Pathogenic | Pathogenic |
| **PolyPhen-2^a^** | Probably damaging | NA^c^ |
| **SIFT^a^** | Tolerated | NA^c^ |
| **MutationTaster** | Disease causing | Disease causing |
| **gnomAD^b^** | Absent | Absent |
| **dbSNP^b^** | Absent | Absent |

1. Pathogenicity threshold: Cut-offs for other prediction tools > 0.5 or no score provided.
2. gnomAD, The Genome Aggregation Database; dbSNP, The Single Nucleotide Polymorphism Database.
3. NA, Not available.
